# Parliament and the Profession

**Published:** 1916-04-29

**Authors:** 


### PARLIAMENT AND THE PROFESSION.

### Health of Munition Workers.

The Minister of Munitions is bearing in mind the importance of the question of the health of munition workers in relation to the financial position of approved societies under the Insurance Act, but, according to a statement made by Dr. Addison, it is not thought necessary to circulate a summary of the memorandum issued by the Health of Munition Workers' Committee as the reports themselves have already been widely circulated to the employers of labour in munition factories.

### Westminster Tuberculosis Dispensary.

The President of the Local Government Board was questioned with regard to the establishment by the Westminster City Council of a tuberculosis dispensary. Negotiations which had been in progress between the Council and the authorities of Charing Cross and St. George's Hospitals having broken down the Council has provided a dispensary of its own at an estimated cost of £1,165 per annum. Major Astor asked what the cost to the ratepayers of the two hospital dispensaries would have been, but beyond stating that the estimated cost of maintenance of the scheme was £1,355 per annum Mr. Long did not supply the House with further information.

### Cowardice of the German Medical Staff.

Arising out of the Report on the Treatment by the Enemy of British Prisoners of War (The Hospital, April 15, p. 57), Mr. Malcolm asked whether any efforts were made by His Majesty's Government to secure an independent medical inspection of the prisoner camp at Wittenberg by the American Ambassador at Berlin or by any of his staff. In reply, Sir Edward Grey stated that the German's permitted no kind of communication, either by letter or orally, as to the condition of Wittenberg, and the Government had no suspicion of the horrors that were going on there, or of the gross and criminal cowardice of the German medical staff in abandoning their duties to those under their charge. "Neither our Allies—whose prisoners in the camp were far more numerous than ours—nor we," added Sir Edward, "demanded independent medical examination of the camp, and on behalf of the Government I can only express profound regret that we so underestimated the brutality of our enemies."

### Treatment of Tuberculous Soldiers.

A good deal of dissatisfaction exists with regard to the method of dealing with soldiers suffering from pulmonary tuberculosis, and there can be no doubt that the system is urgently in need of revision. Under the present arrangement a soldier is discharged from the Army directly it is determined that he is affected by this disease. In the case of other diseases soldiers are given some form of treatment before discharge, and the view held in some quarters is that tuberculous soldiers should not be discharged immediately but should receive the same privileges as other invalid soldiers. This view was expressed by Major Astor in a question he put to the Under Secretary for War, who made the now familiar reply that it was essential that cases of tuberculosis should be specially treated in a sanatorium at as early a date as possible, and that with this object arrangements had been made that soldiers found to be suffering from tubercle of the lung should be discharged the Service at once and admitted to a sanatorium. Mr. Tennant undertook, however, to have an investigation made with a view to ascertaining whether it was desirable to put this disease on all fours with other diseases so as to give soldiers a period of suitable treatment before they are discharged from the Army. The affair is further complicated, first, by the smallness of the accommodation available in sanatoria, and, second, by the fact that there is sometimes great difficulty in persuading these men to go to sanatoria because, as they are discharged from the Army, they get no separation allowance.

